# Adaptation of Chinese and English versions of the Psoriatic Arthritis Quality of Life (PsAQoL) scale for use in Singapore

**DOI:** 10.1186/s12891-016-1292-4

**Published:** 2016-10-18

**Authors:** Ying Ying Leung, Julian Thumboo, Matthew Rouse, Stephen P. McKenna

**Affiliations:** 1 Department of Rheumatology & Immunology, Singapore General Hospital, The Academia, level 4, 20 College Road, Singapore, S169856 Singapore; 2 Duke-NUS Medical School, Singapore, Singapore; 3 Galen Research Ltd, Manchester, UK; 4 School of Health Sciences, University of Manchester, Manchester, UK

**Keywords:** Psoriatic arthritis, Quality of life, outcome measures, cross cultural adaptation

## Abstract

**Background:**

To develop Singapore Chinese and English versions of the Psoriatic Arthritis Quality of Life (PsAQoL) scale that were equivalent to and met the same psychometric and acceptability standards as the original UK measure.

**Methods:**

Translation of the original PsAQoL into contextualised English and Chinese versions for use in Singapore was performed by professional and lay translation panels. Ten Chinese speaking and ten English speaking local patients were interviewed to assess face and content validity. Psoriatic arthritis (PsA) patients (either Chinese or English speaking) fulfilling the Classification criteria of Psoriatic Arthritis were then invited to participate in a validation survey. Clinical variables, the Health Assessment Questionnaire (HAQ) and the Medical Outcome Short-form 36 (SF-36) were used as comparator instruments for convergent validity. A separate sample of PsA patients were invited to participate in a test-retest postal study, with two weeks between administrations.

**Results:**

The validation sample included 98 patients (51 % men) with a mean (SD) age of 51.5 (13.8) years. The PsAQoL had excellent internal consistency (Cronbach’s α = 0.92) and scores on the measure were moderately correlated with health status measures (pain, HAQ score, SF-36 scores) and patient and physician global assessments. The scale was able to distinguish between groups with active or inactive disease assessed by composite scores, HAQ and minimal disease activity. Test–retest reliability was excellent (*r* = 0.92).

**Conclusions:**

This study provides evidence that the adapted English and Chinese versions of the PsAQoL can be used in clinical studies with PsA patients in Singapore.

## Background

Psoriatic arthritis (PsA) is a serious condition that can lead to joint destruction, disability and impaired quality of life (QoL) [1–4]. QoL has been identified as one of the core domains that are important for clinical and observational trials in PsA by the Group for Research and Assessment in Psoriasis and Psoriatic Arthritis (GRAPPA) and has been subsequently endorsed by the Society of Outcome Measures in Rheumatology (OMERACT) [5].

A number of measures have been used to assess patient-reported outcomes (PROs) in PsA. Most of these focus on the symptoms and functional limitations experienced by patients [6–8]. Such measures assess health status rather than QoL. A widely applied model of QoL in health research is the needs-based model [9]. This defines QoL as the extent to which a patient is able to meet his or her fundamental human needs. The model has been applied in the development of over 30 disease-specific PROs, including measures for rheumatoid arthritis [10], ankylosing spondylitis [11], systemic lupus erythematosus [12], osteoarthritis [13] and PsA. These measures have been widely used in multinational clinical trials.

The Psoriatic Arthritis Quality of Life (PsAQoL) scale was the first patient-derived disease-specific instrument for measuring outcome in PsA [14]. It complies with the US Food and Drug Administration (FDA) recommendation of using inputs elicited from patients to support labeling claims [15]. The PsAQoL has been adapted for use in several countries [16, 17] and has been used in clinical trials in PsA [18, 19].

Singapore is a multiethnic Asian country composed of 74.1 % Chinese, 13.4 % Malay, 9.4 % Indian and 3.3 % other ethnicities. Eighty percent of the population are literate in English and 71 % in English and one or more additional languages [20]. Questionnaires available in Chinese and English languages cover 98 % of Singaporeans.

The aim of this study was to develop a version of the PsAQoL suitable for use in Singapore. It was required to meet the same psychometric and acceptability standards as the original United Kingdom (UK) measure. With a resident population of under Six million it is difficult to justify adapting a measure for PsA into the different languages spoken in the country. Consequently, a decision was taken to produce English and Chinese language versions of the PsAQoL and to validate them with a combined sample of patients.

## Methods

All study protocols were approved by the SingHealth Centralized Institutional Review Board. Prior to their inclusion in the study, all patients gave informed consent (CIRB 2012/696/E).

### Translation of the PsAQoL

The PsAQoL was translated into Singapore Chinese and English using the dual-panel methodology, which is used in the adaptation of new language versions of all needs-based QoL measures [21]. Research has shown that the dual-panel methodology produces translations that are more acceptable to patients than the standard forward-backward methodology [22]. The approach involves conducting two independent translation panels; a bilingual panel followed by a lay panel. The purpose of the bilingual panel is to produce an initial translation of the questionnaire into the target language. This version is then presented to a lay panel of monolingual lindividuals of average educational level who assess the items and instructions for comprehensiveness and ‘naturalness’ of language. As the original PsAQoL is in English, only a lay panel was necessary for the Singapore English adaptation, to ensure that the wording of the items was appropriate for local patients. All panels were led by the same moderator.

### Validation of the new language versions

PsA patients fulfilling the Classification criteria of Psoriatic Arthritis (CASPAR) [23] were recruited from a PsA outpatient clinic of a tertiary hospital.

### Cognitive debriefing interviews: field testing for face and content validity

Cognitive debriefing interviews (CDIs) were conducted with both Singapore Chinese speaking and Singapore English speaking PsA patients to test the acceptability, relevance and comprehensiveness of the questionnaire items and instructions. Questionnaires were completed in the presence of an interviewer who made note of any obvious difficulties or hesitation over particular items. Patients were then asked whether they considered the items relevant, applicable and comprehensible and if any important aspects of their experience of PsA had been excluded.

### Psychometric validation

Given the small number of PsA patients in Singapore it was decided to validate the translations with combined samples of Singapore Chinese and English speakers. Two surveys were conducted. The first was a cross-sectional study to determine the internal consistency, convergent validity and known-group validity of the PsAQoL. The second was a postal survey conducted with a separate sample of PsA patients to assess the test-retest reliability of the Singaporean PsAQoL.

### Cross-sectional study

Patients were examined by a rheumatologist for joint and skin condition and Physical Global Assessment (PhGA) was recorded. PsA disease activity was assessed using the clinical Disease Activity Index for Psoriatic Arthritis (cDAPSA) [24], the Composite Psoriatic Disease Activity Index (CPDAI) [25] and Minimal Disease Activity (MDA) [26]. Thresholds are available to stratify patients into high, moderate and low disease activity status. Patients were categorized into four disease activity states by cDAPSA: ≤ 4 (remission); 4 to ≤ 13 (low disease activity); < 13 to ≤ 27 (moderate disease activity) and > 27 (high disease activity). Similarly, three disease activity categories were defined by CPDA: ≤ 3 (mild), 4 to 6 (moderate) and ≥ 7 (severe). The validity, reliability and sensitivity to change of these thresholds have been demonstrated [27, 28].

Patients then completed a demographic questionnaire, the PsAQoL (version dependent on their primary language) and the following comparator scales described below.

#### PsAQoL

The PsAQoL has 20 dichotomous (True/Not true) items. ‘True’ responses are summed to create a total score. High scores indicate poor QoL.

#### The health assessment questionnaire (HAQ) [29]

This measures functional limitations in arthritic disease. It consists of 20 items covering eight categories each with a four-point scale.

#### The short form health survey (SF-36) [30]

This is a generic health status instrument consisting of 36 items which form eight domains (Physical Functioning, Role physical, Bodily pain, General health, Vitality, Social function, Role emotion and Mental health) and two summary scales (Physical Component Summary and Mental Component Summary). Scores for each scale are calculated by summing the items in each domain, which are then transformed onto a scale of 0 ‘worst health’ to 100 ‘best health’. Psychometric evaluation for PsA and using item response theory has been reported [6, 7]. Both English and Chinese language versions of the HAQ and SF-36 have been adapted for use in Singapore [31, 32]. The summary scales were standardized to the population mean (SD) of 50 [10, 33].

Patient global disease activity (PGA) and perceived pain were assessed using a 100 mm visual analogue scale.

### Statistical analyses

Internal consistency measures the extent to which items in a scale are inter-related and is assessed using Cronbach’s alpha coefficient. A value of 0.7 or above indicates that the items are adequately interrelated to form a scale [34].

Convergent validity is determined by assessing the level of association between scores on one scale and those on a comparator scale that measures the same or related constructs. Scores on the PsAQoL were correlated with the clinical assessments and scores on the comparator scales using Spearman’s rank correlation coefficients.

Known group validity is evaluated by testing the ability of a measure to distinguish between groups of people that are known to differ. The factors used for the present study were HAQ score [29], cDAPSA score [24], CPDAI [25] score and MDA [26]. Non-parametric tests for independent samples (Mann–Whitney U test for two groups or Kruskal-Wallis One-Way Analysis of Variance for three or more groups) were employed.

### Test-retest reliability study

The test-retest reliability of a measure is an estimate of its reproducibility over time when no change in condition has taken place. The PsAQoL was administered to patients on two occasions, two weeks apart. This time period was chosen because it is unlikely that disease status will change in two weeks, and this period is long enough to avoid recall bias. The test-retest reliability was assessed by correlating scores on the PsAQoL on two different occasions using Spearman’s rank correlation coefficients. A high correlation indicates that the instrument produces low random measurement error.

## Results

### Translation of the PsAQoL questionnaire

All three translation panels (Chinese bilingual, Chinese lay and English lay) consisted of five participants (two males, three females) aged from 23–72 years. For the Chinese language version, the bilingual panel found the items and instructions straightforward and easy to translate. None of the items required extended discussion.

The Chinese lay panel agreed with most translations provided by the bilingual panel, with the exception of seven items. For example, the item ‘I have to push myself to do things’ was translated as ‘daily household chores’ by the bilingual panel. However, the lay panel thought this may only refer to housework for women and so the item was changed to mean ‘do chores’ in the house.

The Singapore English lay panel found the PsAQoL to be easy to adapt. However, for the item ‘It puts a strain on my personal relationships’, the phrase ‘puts a strain’ was thought to be too difficult for patients to understand. Consensus could not be reached on whether to use ‘affects’ or ‘puts stress on’. Consequently, both versions were assessed in the CDIs.

### Cognitive debriefing interviews: assessment of face and content validity

CDIs were conducted with ten English speaking and ten Chinese speaking PsA patients. Ten men and ten women participated, aged between 29 and 61 years. The mean time taken to complete the questionnaire was 2.8 min.

Overall, patients considered the items and instructions to be clear and easy to understand. There were no particular items that stood out as being awkwardly worded or difficult to understand.

### Psychometric validation

#### Cross-sectional study

Ninety-eight PsA patients took part in the cross-sectional study. Table 1 shows demographic and disease information for the sample. Clinical markers and descriptive statistics for the questionnaires are shown in Table 2. The mean (SD) scores for PsAQoL was 4.5 (5.2). Cronbach’s alpha coefficient for the PsAQoL was 0.92, indicating adequate inter-relatedness of items. Table 3 shows correlations between PsAQoL scores with HAQ scores, SF-36 section scores and various markers of disease activity. PsAQoL scores correlated moderately with the Social functioning, Vitality and General health sections of the SF-36, as well as with HAQ scores and CPDAI, suggesting the importance of these factors to the QoL of PsA patients. There was a very low correlation between PsAQoL scores and two clinical markers; psoriasis area and severity index (PASI) and swollen joint count, suggesting that these have little influence on QoLin PsA.

**Table 1:** Demographic and diseaseinformation of PsA patients (*n* = 98) in the cross-sectional study

Age	Years	
Mean (SD)	51.5 (13.8)	
Duration of PsA		
Mean (SD)	5.5 (8.4)	
Duration of Psoriasis		
Mean (SD)	10.9 (11.7)	
Gender	n	%
Male	50	51.0
Female	48	49.0
Marital Status		
Single	17	17.3
Married	64	65.3
Divorced/Separated	11	11.2
Widowed	4	4.1
Missing	2	2.0
Administered Language		
English	66	67.3
Chinese	32	32.7
Ethnicity		
Chinese	65	66.3
Malay	5	5.1
Indian	23	23.5
Other	5	5.1
Education		
Primary or Below	19	19.4
Secondary	39	39.8
Post-Secondary	18	18.4
Tertiary	21	21.4
Missing	1	1.0
cDAPSA		
Remission	20	20.4
Low disease activity	47	48.0
Moderate disease activity	22	22.4
High disease activity	6	6.1
Missing	3	3.1
CPDAI		
Mild	35	35.7
Moderate	28	28.6
Severe	22	22.4
Missing	13	13.3
Minimal Disease Activity	38	38.2

*PsA* psoriatic arthritis, *cDAPSA* clinical disease activity in psoriatic arthritis score, *CPDAI* composite psoriatic disease activity index

cDAPSA: ≤ 4 (remission); 4–≤ 13 (low disease activity); < 13–≤ 27 (moderate disease activity) and > 27 (high disease activity)

CPDAI: ≤ 3 (mild), 4–6 (moderate) and ≥ 7 (severe)

**Table 2:** Clinical markers in the Cross-sectional study (*n* = 98)

	Mean (SD)
Clinically damaged joint count (0–68)	5.0 (9.3)
Tender joint count (0–68)	2.9 (4.0)
Swollen joint count (0–66)	2.0 (2.7)
Dactylitis count (0–20)	0.8 (1.4)
Leeds Enthesitis Index (0–6)	0.3 (0.6)
PASI (0–72)	3.3 (6.0)
Pain (0–100)	33.4 (25.9)
Patient Global Assessment (0–100)	33.1 (23.5)
Physician Global Assessment (0–100)	2.9 (1.8)
ESR, mm/h	26.1 (21.6)
cDAPSA (0–154)	11.5 (9.3)
CPDAI (0–15)	5.1 (3.0)
HAQ (0–3)	0.4 (0.6)

*PASI* psoriasis area and severity index, *ESR* erythrocyte sedimentation rate, *HAQ* Health Assessment Questionnaire, *cDAPSA* clinical Disease activity in psoriatic arthritis score, *CPDAI* composite psoriatic disease activity index

**Table 3:** Association between PsAQoL scores and measures of disease activity and health status

	PsAQoL
Pain	0.51*
Tender joint count	0.35*
Swollen joint count	0.16
PASI	0.10
Patient global assessment	0.47*
Physician global assessment	0.43*
cDAPSA	0.48*
CPDAI	0.55*
HAQ	0.57*
SF36	
Physical Functioning	−0.42*
Role Physical	−0.48*
Bodily Pain	−0.50*
General Health	−0.58*
Vitality	−0.58*
Social Functioning	−0.62*
Role Emotional	−0.44*
Mental Health	−0.53*
Physical Component Summary	−0.51*
Mental Component Summary	−0.62*

*p < 0.0001

*PASI* psoriasis area and severity index, *cDAPSA* clinical Disease activity in psoriatic arthritis score, *CPDAI* composite psoriatic disease activity index, *HAQ* Health Assessment Questionnaire, *SF36* Medical Outcome Short Form 36

**Fig. 1:**
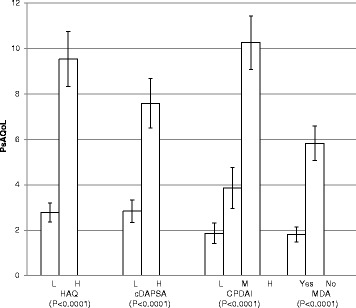
Mean PsAQoL scores stratified by functional limitation or disease activity. HAQ = health assessment questionnaire; cDAPSA = clinical disease activity in psoriatic arthritis score; CPDAI = composite psoriatic disease activity index; MDA = minimal disease activity. L = Low disease activity. M = moderate disease activity. H = high disease activity

Figure 1 shows mean PsAQoL scores grouped by HAQ score, cDAPSA score, CPDAI score and MDA. Disease activity measured by cDAPSA was dichotomized into high (moderate and high) versus low disease activity (remission and low disease activity). Due to the distribution of responses to the item. Significant differences were found between patients grouped by these factors, demonstrating the ability of the PsAQoL to distinguish between subgroups of patients (all p-values < 0.0001).

### Test-retest reliability study

A separate sample of 38 PsA patients completed the PsAQoL on two separate occasions, approximately two weeks apart. The sample consisted of 17 (44.7 %) men and 21 (55.3 %) women, with a mean (SD) age of 53.9 (11.5) years. Twenty (52.6 %) patients completed the Singapore Chinese version and 18 (47.4 %) completed the English version. The mean (SD) PsAQoL scores at baseline and week two were 2.7 (4.2) and 2.4 (4.4), respectively. The Spearman’s rank correlation coefficienct was 0.92, indicating the excellent reproducibility of the instrument. This means that the PsAQoL has a very low level of error measurement (6 %).

## Discussion

The Singapore Chinese and English versions the PsAQoL were translated and culturally adapted to make them suitable for use in Singapore and were found to be of a comparable quality to the original UK instrument. Patients found the measure to be relevant, understandable and easy to complete in only a few minutes. Good internal consistency, reproducibility and construct validity were demonstrated. The Singapore PsAQoL was able to distinguish between groups that differed by PsA activity level, remission and functional status.

The PsAQoL is the first PsA-specific instrument for measuring QoL and has been adapted for use in 48 languages [16, 17]. This is the first study to translate and adapt the PsAQoL into Chinese. While spoken Chinese has many dialects, written Chinese is in the same format. Consequently, disparate dialect groups are able to communicate through writing [35]. As Chinese is spoken by nearly 1.3 billion people (approximately 16 % of the world's population) [36], the adaptation of PsAQoL into Chinese will greatly increase the reach of the instrument for the measurement of QoL in PsA patients. The usage of specific Chinese words may vary among Chinese speakers in different geographic region, further adaptations in different region could be achieved via patient debriefing sessions.

In this study, we simultaneously adapted the PsAQoL into Singapore English and Singapore Chinese. English is the official language in Singapore, and most people in Singapore are bilingual in at least two languages [20]. In daily clinical practice and clinical trials in the local environment, patients are allowed to choose their own language of choice for PROs. Therefore, we think the simultaneous validation process is practical and appropriate to the local setting. Ideally, the absence of bias from languages should be demonstrated by the application of Rasch analysis but this requires a larger sample size.

PsAQoL scores in the present study were low compared to scores in the PsAQoL development study in the UK [14] but similar to those found in the Swedish and Dutch studies [16, 17]. In all adaptation studies, 20–30 % of patients scored the minimum and between one and two percent scored the maximum. The low scores on the PsAQoL in this study probably reflect the relatively mild disease in the sample studied.

The main limitation of the study is the small sample size, which reflects the size of the Singapore population. This prevented an investigation of the comparability of the performance of the Chinese and English versions. Versions of measures are easily adapted from UK English to other countries where English is spoken [16, 17] and there is also the potential to combine the Chinese speaking population in Singapore from that in other countries. This would then allow the performance of the two language versions to be compared. Secondly, we stratified disease activity using patient-reported instruments, which may be affected by comorbidities such as depression and fibromyalgia. Instruments that incorporate C-reactive protein (CRP), like (PASDAS) [37] and DAPSA may reflect disease activity more objectively. However, as CRP was not included as a routine test in the clinic at the initiation of the study, only 30 % of patients could have a PASDAS and DAPSA score, and therefore not reported in this study. Finally, this cross-sectional study was not designed to assess the sensitivity to change of the PsAQoL.

## Conclusions

In conclusion, the present study showed that the Singapore Chinese and English versions of the PsAQoL are valid and reliable instruments when used with combined samples of PsA patients who speak either of these languages. Patients found the instrument relevant to their perspective, easy to understand and complete and easy to administer. The study provides evidence that these versions of PsAQoL can be used in clinical and observational studies for PsA patients in Singapore.

## Abbreviations

CASPAR: Classification criteria of Psoriatic Arthritis
cDAPSA: clinical disease activity index for psoriatic arthritis
CDIs: Cognitive debriefing interviews
CPDAI: Composite psoriatic disease activity index
FDA: US food and drug administration
GRAPPA: Group for research and assessment in psoriasis and psoriatic arthritis
HAQ: Health assessment questionnaire
MDA: Minimal disease activity
OMEARCT: Outcome measures in rheumatology
PASI: psoriasis area and severity index
PGA: Patient global disease activity
PhGA: Physical global assessment
PROs: patient-reported outcomes
PsA: Psoriatic arthritis
PsAQoL: Psoriatic arthritis quality of life scale
QoL: Quality of life
SF-36: Medical outcome short-form 36
UK: United Kingdom
